# Ontogenetic variation in rat liver, lung and kidney monooxygenase induction by low doses of benzo(A)pyrene and cigarette-smoke condensate.

**DOI:** 10.1038/bjc.1981.290

**Published:** 1981-12

**Authors:** J. van Cantfort, J. E. Gielen

## Abstract

The specific lung-AHH induction, which we previously observed after the inhalation of cigarette smoke, is not due to the route followed by the inhaled smoke, for the same phenomenon occurs after i.p. injection of either cigarette smoke condensate (CSC) or benzo(a)pyrene in low doses. In this respect lung AHH behaves completely differently from the liver and kidney enzyme, in which organs, basal AHH activity (which is low in the foetus) increases rapidly after birth to reach the adult level 2 months later, and is only inducible by CSC and low doses of BP in unweaned rats. In the lung, the basal AHH activity (low in the foetus) increases abruptly at birth, peaks in 5-day-old rats and then decreases slightly. Contrary to enzyme activity in other tissues, lung AHH cannot be induced in unweaned young animals. The enzyme subsequently becomes sensitive to inducing agents and is highly inducible in 90-day-old rats. Similar behaviour occurs in 2 other enzymes linked to cytochrome P1450: ethoxycoumarin deethylase and ethoxyresorufin deethylase. The results could be related to the particular susceptibility of the lung to develop cancer after the inhalation of cigarette smoke.


*Br. J. Cancer* (1981) **44**, 902

### ONTOGENETIC VARIATION IN RAT LIVER, LUNG AND KIDNEY MONOOXYGENASE INDUCTION BY LOW DOSES OF BENZO(A)PYRENE AND CIGARETTE-SMOKE CONDENSATE

J. VAN CANTFORT AND J. E. GIELEN*

*From the Laboratoire de Chimie Médicale, Institut de Pathologie, Université de Liège, Bâtiment B 23, Belgium*

Received 1 June 1981 Accepted 20 August 1981

**Summary.—The specific lung-AHH induction, which we previously observed after the inhalation of cigarette smoke, is not due to the route followed by the inhaled smoke, for the same phenomenon occurs after i.p. injection of either cigarette smoke condensate (CSC) or benzo(a)pyrene in low doses. In this respect lung AHH behaves completely differently from the liver and kidney enzyme, in which organs, basal AHH activity (which is low in the foetus) increases rapidly after birth to reach the adult level 2 months later, and is only inducible by CSC and low doses of BP in unweaned rats.**

**In the lung, the basal AHH activity (low in the foetus) increases abruptly at birth, peaks in 5-day-old rats and then decreases slightly. Contrary to enzyme activity in other tissues, lung AHH cannot be induced in unweaned young animals. The enzyme subsequently becomes sensitive to inducing agents and is highly inducible in 90-day-old rats.**

**Similar behaviour occurs in 2 other enzymes linked to cytochrome $P_1450$: ethoxycoumarin deethylase and ethoxyresorufin deethylase. The results could be related to the particular susceptibility of the lung to develop cancer after the inhalation of cigarette smoke.**

MOST CHEMICAL CARCINOGENS are harmless lipophilic molecules which cannot be eliminated from the body unless they are transformed into more polar, hydrosoluble metabolites. This transformation is catalysed by the successive action of microsomal monooxygenases, epoxide hydrolase and conjugating enzymes such as sulphotransferase, UDP-glucuronyl transferase or glutathione transferase (Heidelberger, 1975; Miller & Miller, 1976; Weisburger, 1978; Gielen, 1978).

The first enzymic reaction of the polycyclic-hydrocarbon metabolic pathway is catalysed by a cytochrome P450-dependent monooxygenase, the aryl hydrocarbon hydroxylase (AHH). This leads to the formation of reactive arene oxides, which are further metabolized by enzymic and non-enzymic reactions into many metabolites (DePierre & Ernster, 1978). These reactive intermediates could also bind covalently to cellular macromolecules, and generate a variety of toxic effects inducing mutations and cancer (Heidelberger, 1975; Miller & Miller, 1976; Weisburger, 1978; Nebert & Jensen, 1979). To measure the activity of the AHH, it is necessary to quantitate all the metabolites, as they are all derived from the arene oxides initially produced by the AHH. This assay can only be performed accurately by an isotopic method which uses [$^3$H]-benzo(a)pyrene as a substrate of the enzyme reaction (Van Cantfort *et al.*, 1977).

Cigarette smoke appears to be one of the commonest carcinogenic products of our environment. Numerous epidemiological studies have shown an increase in the

* To whom all correspondence should be addressed.

incidence of cancer in several organs, notably the lung, among cigarette smokers (Wynder & Mabuchi, 1972; Doll, 1977; 1978). This phenomenon has been experimentally reproduced in several animals or *in vitro* models (Akin *et al.*, 1976; Kouri *et al.*, 1979; Florin *et al.*, 1980).

This particular susceptibility of the lung to initiate cancer on exposure to cigarette smoke could be due to several factors: (a) substances in cigarette smoke which induce monooxygenases; (b) the direct action of toxic components in smoke on the first organ reached; (c) numerous potentially carcinogenic agents in smoke which can be activated into highly reactive metabolites; (d) the very low activity of lung conjugating enzymes which catalyse the elimination of these active metabolites (Ball *et al.*, 1979).

The inducing effect of cigarette smoke is the best known phenomenon. In man, this inducing action of cigarette smoke has been demonstrated in placentas collected at birth from smoking and non-smoking women (Nebert *et al.*, 1969; Vaught *et al.*, 1979) and in the pulmonary alveolar macrophages of smokers and non-smokers (Cantrell *et al.*, 1973). In rats, mice and hamsters, AHH is specifically induced by cigarette smoke in the lung and kidney (Abramson & Hutton, 1975; Welch *et al.*, 1971; Dansette *et al.*, 1979; Bilimoria & Ecobichon, 1980; Van Cantfort & Gielen, 1977). In both tissues, the maximal effect has an induction factor of 10, but the lung enzyme is much more sensitive, since it demonstrates a greater response to small doses of cigarette smoke (Van Cantfort & Gielen, 1977). This induction of monooxygenases leads to the formation of more active metabolites when the lung is treated with [$^3$H]-benzo(a)pyrene, and an increase in its covalent binding to macromolecules (Cohen *et al.*, 1977).

On the other hand, cigarette smoke has no action on AHH activity in the liver (Abramson & Hutton, 1975; Dansette *et al.*, 1979; Van Cantfort & Gielen, 1977). This organotropism can be explained by the particular route followed by cigarette-smoke components, which, after inhalation, first reach the lung where their concentration is obviously the highest. After absorption into the pulmonary blood, they go directly to the left side of the heart and to the kidney where they are filtered before reaching other tissues such as the liver.

In this paper, we show that this organotropism is also due to the particular sensitivity of extrahepatic tissues to the inducing action of the polycyclic hydrocarbons in cigarette smoke. Parallel induction can be obtained by inhaling cigarette smoke and by i.p. injection of cigarette-smoke condensate (CSC), or low doses of BP. We thus demonstrate that three parameters are important: the concentration of the polycyclic hydrocarbons administered, the age of the animal, and the organ investigated.

### MATERIAL AND METHODS

Benzo(a)pyrene was purchased from Fluka (New-Ulm, F.R.G.). Aldrin and dieldrin were from Riedel-de-Haen (Hannover, F.R.G.); glucose-6-phosphate dehydrogenase and enzymic co-factors were from Boehringer (Mannheim, F.R.G.). CSC was a generous gift from Dr R. E. Kouri (Microbiological Associates, Bethesda, Maryland, U.S.A.). Other chemicals and solvents were of analytical grade and were obtained from Merck (Darmstadt, F.R.G.). $^3$H-benzo(a)pyrene (1·2 Ci/mmol) was purchased from I.R.E. (Fleurus, Belgium) and $^3$H-benzo(a)pyrene 4·5 oxide (2·3 Ci/mmol) was prepared according to the method of Dansette & Jerina (1974).

*Treatment of animals.*—All the experiments were performed on Sprague–Dawley rats obtained from the Centre des Oncins (Lyon, France). They were kept at a constant temperature of 22°C with free access to food (UAR A03, Villemoisson, France) and tap water.

BP and CSC, dissolved in dimethylsulphoxide, were given by 3 successive i.p. injections at 24h intervals for 3 consecutive days. Control animals received the same quantity of the vehicle only (0·5 ml/kg).

The animals were killed by decapitation 24 h after the last injection. The organs were removed and immediately plunged in cold isotonic KCl. Livers, kidneys and lungs were then pressed through a metallic disc perforated with 1·5mm-diameter holes to eliminate the connective tissues. For each organ, the resulting pulp was diluted with 4 parts of a Tris (0·01M, pH 7·4) sucrose (0·25M) buffer and homogenized in a Potter Elvejhem tube with a teflon pestle. The homogenate was centrifuged for 10 min at 10,000 *g* in a refrigerated Sorvall (RC 2B) and the supernatant, stored at −20°C, was used as a source for enzyme analysis.

*Enzyme assays.*—AHH activity was measured by an isotope method developed in our laboratory (Van Cantfort *et al.*, 1977; Manil *et al.*, 1981). The measurement of aldrin epoxidase was performed by the method described by Wolff *et al.* (1979). The assays for ethoxycoumarin deethylase (Aitio, 1978), ethoxyresorufin deethylase (Burke & Mayer, 1974), aminopyrine-N-demethylase (Christensen & Wissig, 1972), epoxide hydrolase (Schmassman *et al.*, 1976), and epoxide–glutathione transferase (Van Cantfort *et al.*, 1979) have also been described. In each case, we used the Lowry method to verify that the treatment did not influence the protein concentration of the organ.

FIG. 1.—Effect of i.p. injection of cigarette smoke condensate on AHH activity in rat liver, lung and kidney. AHH was measured in the 9000*g* supernatant of liver, kidney and lung homogenates obtained from male Sprague–Dawley rats (250 g) treated with the vehicle alone (100 μl DMSO; Co) or with 5, 20 and 50 mg/kg of cigarette smoke condensate (CSC). Three successive administrations were performed at 24h intervals the rats were killed on the 4th day. The results correspond to the mean ± s.d. (vertical bars) from 5 rats. * = $P < 0{\cdot}05$ compared to control animals.

### RESULTS

### *Specific lung and kidney AHH induction after i.p. injection of cigarette-smoke condensate*

We previously demonstrated that cigarette-smoke inhalation specifically induces AHH in the lung and kidney, but has no effect on the liver enzyme (Van Cantfort & Gielen, 1977). In order to demonstrate the importance of the method of administering the smoke on the specific induction of AHH in extrahepatic organs, we studied the effect of i.p. injection of CSC.

When administered i.p., the chemicals obviously reached the liver before the other organs. Nevertheless, the lung was surprisingly the most sensitive organ, followed by the kidney and then the liver (Fig. 1). With a 5mg/kg dose, only the lung AHH activity was significantly induced. With a 20mg/kg dose, both the lung and kidney enzymes were induced, but only a 50mg/kg dose induced the liver enzyme. These results thus repeated the effects of the inhalation of cigarette smoke. We thus concluded that the CSC contained substances with a selective action on extrahepatic tissues, and notably, the lung or that the lung was the most sensitive tissue to the inducers in CSC.

### *Ontogenetic variation in liver, kidney and lung AHH induction after i.p. injections of CSC*

As shown in Figs 2, 3 and 4, AHH inducibility varied greatly during the ontogeny of the animals and was quite different in the lung compared to the kidney and liver.

In the liver, CSC, either at a 5mg/kg or 25mg/kg dose, did not induce AHH in the foetus and in 5-day-old rats. Surprisingly, in 15-day-old animals, CSC was a very good inducer of AHH, but this property rapidly disappeared in older animals, and was never observed in rats more than 30 days old (data not shown).

In the kidney, a similar phenomenon occurred (Fig. 2). AHH was not induced

FIG. 2.—Effect of i.p. injection of CSC on rat kidney AHH activity as a function of age. Rats were treated 3 times at 24h intervals with CSC (5 or 25 mg/kg) and killed on the 4th day. AHH was measured in the 9000*g* supernatant. The results show mean ± s.d. from 3 pools of 5 rats (5 and 15 days old), 5 pools of 2 rats (30 days old) and 5 individual rats (60 and 90 days old). *$P < 0{\cdot}05$.

after i.p. injection of CSC in the foetus or in very young animals. It was well induced in 15-day-old rats, especially by the larger dose, but contrary to the liver, AHH remained slightly inducible by the 25mg/kg dose in the older animals.

In the lung, this ontogenetic variation of AHH inducibility was quite different (Fig. 3). AHH was insensitive to the action of CSC in unweaned animals. Its inducibility then continuously increased with age. In fact, from the 15th to the 90th day after birth, the basal AHH activity in control rats continuously decreased, but the activity induced by a 25mg/kg dose, gradually *increased.* In 3-month-old rats, the induction factor thus increased to 4. In older animals, both basal and induced AHH activities remained constant, as similar results were obtained from 9-month-old rats (data not shown).

FIG. 3.—Effect of i.p. injection of CSC on rat lung AHH activity as a function of age. Methodological details as in Fig. 2.

*Ontogenetic variation in liver, kidney and lung AHH induction after i.p. injection of BP*

As polycyclic hydrocarbons are generally considered to be the most powerful inducers in cigarette smoke, we tested the effect of one of the main members of this class: benzo(a)pyrene, though BP is seldom used in induction experiments *in vivo*; preference usually being given to 3-methylcholanthrene or dimethylbenzanthracene, which have a much more inducing and carcinogenic potential (Buty *et al.*, 1976).

In our study, BP was injected i.p. into the animals, either at a low dose (1 mg/kg) or at a higher dose (80 mg/kg) which was known to produce an optimal induction in most of the tissues tested (Ciaccio & De Vera, 1976).

FIG. 4.—Influence of age on the induction of AHH in the liver by low (1 mg/kg, ○) or high (80 mg/kg, ■) doses of BP. (Controls, ●). For experimental procedures, see legend to Fig. 2.

FIG. 5.—Influence of age on the induction of AHH in the kidney by low (1 mg/kg, ○) or high (80 mg/kg, ■) doses of BP. (Controls, ●.) For experimental procedures, see legend to Fig. 2.

In the rat liver, AHH activity was highly induced by the larger BP dose, regardless of the age of the animal; with lower doses, hepatic AHH activity was only significantly induced in 15-day-old animals (Fig. 4).

FIG. 6.—Influence of age on the induction of AHH in the lung by low (○) or high (■) doses of BP (Controls, ●). For experimental procedures, see legend to Fig. 2.

The phenomenon in the kidney was identical: a high dose of BP induced AHH activity, whatever the age of the animal. On the other hand, the enzyme was only induced by a low dose of BP in young, unweaned rats. In 15-day-old animals, we even observed induction by a factor of 4, but this effect rapidly diminished in older rats (Fig. 5).

As with CSC, lung AHH responded to BP injections entirely differently. Young rats were nearly insensitive to BP injections, even at 80mg/kg doses, and AHH inducibility increased with age to reach a maximum in the adult animal. Low doses of BP were then nearly as efficient in inducing AHH activity as the 80mg/kg dose. In 9-month-old rats, lung AHH basal activity decreased, but its inducibility remained very pronounced (Fig. 6).

*Ontogenetic variation in the induction of ethoxycoumarin deethylase in the liver, and in the induction of hepatic and extrahepatic ethoxyresorufin deethylase after i.p. injection of BP*

As extrahepatic AHH belongs to the class of monooxygenases with cytochrome $P_1450$ or P448 as a terminal oxidase, we tested the action of BP on 2 other microsomal monooxygenases linked to this type of cytochrome; *viz.* ethoxycoumarin deethylase and ethoxyresorufin

FIG. 7.—Influence of age on the induction of ethoxycoumarin deethylase (A) and ethoxyresorufin deethylase (B) in the liver by low (○) or high (■) doses of BP (Controls, ●). For experimental procedures, see legend to Fig. 2.

FIG. 8.—Influence of age on the induction of ethoxyresorufin deethylase in the kidney and lung by low (○) or high (■) doses of BP (Controls, ●). For experimental procedures, see legend to Fig. 2.

deethylase. These enzymes metabolize a drug which is not a polycyclic hydrocarbon. As shown in Fig. 7, these enzymes are induced only in 15-day-old rat liver by low doses of BP, whereas the induction of these enzymes by high doses of BP was possible at any age.

Unfortunately, in other tissues, ethoxycoumarin deethylase cannot be measured by our technique; only results from ethoxyresorufin deethylase assays can be presented.

In the kidney and lung, ethoxyresorufin deethylase displays an inducibility that is comparable to that of AHH (Fig. 8). In the kidney, low-dose BP induces ethoxyresorufin deethylase activity only in 15-day-old rats. In the lung, the inducibility increases with age; it does not exist in 15-day old rats, is 3-fold in 30-day-old rats, and 5-fold in 3-month-old rats. In all cases, the 1mg/kg dose is as efficient as the higher dose, both producing the same effect.

*Enzymes not induced by i.p. injections of BP or CSC*

We tested the effect of the same dose (25 mg/kg) of either BP or CSC on some enzymes currently assayed in our laboratory. This included microsomal monooxygenases linked to cytochrome P450, such as aldrin epoxidase, aminopyrine-N-demethylase and benzphetamine-N-demethylase, as well as epoxide hydrolase and epoxide-glutathione transferase, which are both involved in the metabolism of polycyclic hydrocarbons. All these enzymes could be measured in the liver, kidney and lung (with some difficulties for the demethylases in extrahepatic tissues). Nevertheless, none was modified by BP or CSC, regardless of the tissue analysed (data not shown).

*Organ weight and protein content at various ages*

Throughout this paper, AHH activities are expressed in units per gram of tissue. However, any other unit (*e.g.* per mg protein) will give qualitatively similar results, as protein content is hardly influenced by the treatment or the age of the animals (data not shown).

### DISCUSSION

Numerous studies on rats (Welch *et al.*, 1971; Van Cantfort & Gielen, 1977) and mice (Abramson & Hutton, 1975; Van Cantfort & Gielen, 1975; Kouri *et al.*, 1974) have demonstrated that cigarette smoke contains potent inducing agents of lung and kidney AHH activity. Nevertheless, given the dose inhaled, the kinetics of the AHH induction and the organotropism, we previously postulated that polycyclic hydrocarbons linked to smoke could not be the main AHH-inducing agents (Van Cantfort & Gielen, 1977). Kouri *et al.* (1974) demonstrated that it was necessary to administer at least 10 mg/kg of methylcholanthrene via the trachea to induce pulmonary AHH in the mouse, *i.e.* a dose which is 5000× that of CSC to give the same effect.

The experiments described in this paper do not confirm this hypothesis, but on the contrary, lead us to believe that the action of low doses of polycyclic hydrocarbons explains the induction observed after the inhalation of cigarette smoke. I.p. injection of CSC selectively induces pulmonary and renal AHH in a manner similar to that of the inhalation of smoke. This experimental model is particularly easy to control, and it is much easier to establish quantitative comparisons. Such a study conducted on a small number of animals (data not shown) shows that similar inductions of the AHH activity are obtained after i.p. injections of BP (0·1 mg/kg) or CSC (25 mg/kg). According to Akin *et al.* (1976), 1 g of CSC contains $\sim 200\ \mu g$ of polycyclic hydrocarbons and 5 $\mu g$ of BP. Thus, the total amount of polycyclic hydrocarbons linked to CSC is one-twentieth the amount of BP required to produce the same induction. This quantitative difference can be explained in several ways: (a) the existence of more powerful inducers than the polycyclic hydrocarbons in the CSC: (b) the existence of polycyclic hydrocarbons which are more powerful inducers than BP; (c) a synergic effect of CSC constituents. The parallel inducing effects of CSC and BP as a function of the age of the animal and the organ under investigation are an additional biological argument for the hypothesis that polycyclic hydrocarbons play an important part in the inducing action of CSC.

The age-related variation of the BP induction of AHH activity in various organs is surprising. In the liver and kidney, the enzyme is inducible by a low dose of BP only during a short period before weaning. These results must be interpreted on the basis of the multiplicity or specificity of microsomal monooxygenases (Ullrich & Kremers, 1977; Lu & Levin, 1974; Nebert, 1979). These enzymes exist as different molecular forms having variable specificities towards substrates or the position on these substrates (Nebert & Jensen, 1979). They metabolize pesticides and exogenous drugs, as well as steroids or endogenous fatty acids (Nebert & Jensen, 1979). Hence, the temporary AHH inducibility during the development of the animal could correspond to the appearance of monooxygenases with a physiological role in the maturation of the organism during this period. This observation supports that of Atlas *et al.* (1977) who demonstrated the existence of a temporal control of the monooxygenases linked to P450 and $P_1450$ in the rabbit.

The non-induction by low doses of CSC and BP in the adult liver and kidney could be explained by the presence of inducers in the diet which would make the enzyme refractory to additional induction. This hypothesis would be difficult to reconcile with the following observations: (1) the kidney and liver AHH activities were well induced by higher doses of BP in the adult animal and (2) the basal activity of the lung enzyme decreased with the age of the rat and responded to the lowest doses of the inducers.

In the lung and with respect to carcinogenesis, the increase in inducibility in adult animals could correspond to a higher risk factor. Moreover, the great sensitivity of the lung to low doses of BP should be related to the increase in the development of lung cancer in smokers.

We believe that the special AHH inducibility of the lung of the adult animal is a very important biological fact; our forthcoming studies should give us insight into the cause and biological consequences of this phenomenon.

This work was financially supported by Grant 1072 from the Council for Tobacco Research—U.S.A., Inc. and by Grants 39001.79, 34522.81, 34523.81, and 35424.81 from the Fonds de la Recherche Scientifique Médicale. The authors are grateful to Ms Martine Verbys, Ms Janice Lynn Delaval and Ms Marie-Thérèse d'Arripe for their help in the preparation of this manuscript. The authors also acknowledge the technical assistance of Ms Micheline Poma-Léonard and Ms Jocelyne Doyen-Sele.

### REFERENCES

Abramson, R. K. & Hutton, J. J. (1975) Effects of cigarette smoking on aryl hydrocarbon hydroxylase activity in lungs and tissues of inbred mice. *Cancer Res.*, **35**, 23.

Aitio, A. (1978) A simple and sensitive assay of 7-ethoxycoumarin deethylation. *Analyt. Biochem.*, **85**, 488.

Akin, F. J., Snook, M. E., Severson, R. E., Chamberlain, W. J. & Walters, D. B. (1976) Identification of polynuclear aromatic hydrocarbons in cigarette smoke and their importance as tumorigens. *J. Natl Cancer Inst.*, **57**, 191.

ATLAS, S. A., BOOBIS, A. R., FELTON, J. S., THORGEIRSSON, S. S. & NEBERT, D. W. (1977) Ontogenetic expression of polycyclic aromatic compound-inducible monooxygenase activities and forms of cytochrome P-450 in rabbit. Evidence for temporal control and organ specificity of two genetic regulatory systems. *J. Biol. Chem.*, **252**, 4712.

BALL, L. M., PLUMMER, J. L., SMITH, B. R. & BEND, J. R. (1979) Benzo(a)pyrene oxidation, conjugation and disposition in the isolated perfused rabbit lung: Role of the glutathione S-transferases. *Med. Biol.*, **57**, 298.

BILIMORIA, M. H. & ECOBICHON, D. J. (1980) Responses of rodent hepatic, renal and pulmonary aryl hydrocarbon hydroxylase following exposure to cigarette smoke. *Toxicology*, **15**, 83.

BURKE, M. D. & MAYER, R. T. (1974) Ethoxyresorufin: Direct fluorimetric assay of a microsomal O-dealkylation which is preferentially inducible by 3-methylcholanthrene. *Drug Metab. Dispos.*, **2**, 583.

BUTY, S. G., THOMPSON, S. & SLAGA, T. J. (1976) The role of epidermal aryl hydrocarbon hydroxylase in the covalent binding of polycyclic hydrocarbon to DNA and its relationship to tumor initiation. *Biochem. Biophys. Res. Commun.*, **70**, 1102.

CANTRELL, E. T., WARR, G. A., BUSBEE, D. L. & MARTIN, R. R. (1973) Induction of aryl hydrocarbon hydroxylase in human alveolar macrophages by cigarette smoking. *J. Clin. Invest.*, **52**, 1881.

CHRISTENSEN, F. & WISSIG, F. (1972) Inhibition of microsomal drug metabolizing enzymes from rat liver by various 4-hydroxycoumarin derivatives. *Biochem. Pharmacol.*, **21**, 975.

CIACCIO, E. I. & DE VERA, H. (1976) Effect of benzo(a)pyrene and chlorpromazine on aryl hydrocarbon hydroxylase activity from rat tissues. *Biochem. Pharmacol.*, **25**, 985.

COHEN, G. M., UOTILA, P., HARTIALA, J., SUOLINNA, E. M., SIMBERG, N. & PELKONEN, O. (1977) Metabolism and covalent binding of [$^3$H]-benzo(a)pyrene by isolated perfused lungs and short-term tracheal organ culture of cigarette smoke-exposed rats. *Cancer Res.*, **37**, 2147.

DANSETTE, P. M. & JERINA, D. M. (1974) A facile synthesis of arene oxides at the K-regions of polycyclic hydrocarbons. *J. Am. Chem. Soc.*, **96**, 1224.

DANSETTE, P. M., ALEXANDROV, K., AZERAD, R. & FRAYSSINET, CH. (1979) The effect of some mixed function oxidase inducers on aryl hydrocarbon hydroxylase and epoxide hydratase in nuclei and microsomes from rat liver and lung. The effect of cigarette smoke. *Eur. J. Cancer*, **15**, 915.

DEPIERRE, J. W. & ERNSTER, L. (1978) The metabolism of polycyclic hydrocarbons and its relationship to cancer. *Biochim. Biophys. Acta*, **473**, 149.

DOLL, R. (1977) Incidence of cancer in humans. In *Origins of Human Cancer*, Book A. Ed. Hiatt *et al.* U.S.A.: Cold Spring Harbor Lab. p. 1.

DOLL, R. (1978) An epidemiological perspective of the biology of cancer. *Cancer Res.*, **38**, 3573.

FLORIN, I., RUTBERG, L., CURVALL, M. & ENZELL, C. R. (1980) Screening of tobacco smoke /constituents for mutagenicity using the Ames' test. *Toxicology*, **18**, 219.

GIELEN, J. E. (1978) Biochemical aspects of chemical carcinogenesis. *Bull. Cancer*, **65**, 249.

HEIDELBERGER, C. (1975) Chemical carcinogenesis. *Ann. Rev. Biochem.*, **44**, 79.

KOURI, R. E., DEMOISE, C. F. & WHITMIRE, C. E. (1979) The significance of aryl hydrocarbon hydroxylase enzyme systems in the selection of model systems for respiratory carcinogenesis. In *Experimental Lung Cancer: Carcinogenesis and Bioassays.* Eds Karbe & Park. New York: Springer Verlag. p. 48.

KOURI, R. E., RUDE, T. H., CURRAND, R. D., BRAND, K. R., SASNOWSKI, R. E., SCHECHTMAN, L. M., BENEDICT, W. F. & HENRY, C. H. (1979) Biological activity of tobacco smoke and tobacco smoke-related chemicals. *Environ. Hlth Perspect.*, **29**, 63.

LU, A. Y. H. & LEVIN, W. (1974) The resolution and reconstitution of the liver microsomal hydroxylation system. *Biochim. Biophys. Acta*, **344**, 205.

MANIL, L., VAN CANTFORT, J., LAPIERE, CH. M. & GIELEN, J. E. (1981) Significant variations of mouse skin AHH inducibility as a function of the hair growth cycle. *Br. J. Cancer*, **43**, 210.

MILLER, E. C. & MILLER, J. A. (1976) The metabolism of chemical carcinogens to reactive electrophiles and their possible mechanism of action in carcinogenesis. In: *Chemical Carcinogens.* Ed. Searle. Washington: ALS Monograph. p. 737.

NEBERT, D. W. (1979) Multiple forms of inducible drug-metabolizing enzymes: A reasonable mechanism by which any organism can cope with adversity. *Mol. Cell. Biochem.*, **27**, 27.

NEBERT, D. W., WINKER, J. & GELBOIN, H. V. (1969) Aryl hydrocarbon hydroxylase activity in human placenta from cigarette smoking and non-smoking women. *Cancer Res.*, **29**, 1763.

NEBERT, D. W. & JENSEN, N. M. (1979) The Ah locus: Genetic regulation of the metabolism of carcinogens, drugs and other environmental chemicals by cytochrome P450 mediated monooxygenases. In *Critical Reviews in Biochemistry.* Cleveland: CRC Press Inc., Ohio. p. 401.

SCHMASSMAN, H. U., GLATT, H. R. & OESCH, F. (1976) A rapid assay for epoxide hydratase activity with benzo(a)pyrene-4,5-(K-region) oxide as substrate. *Analyt. Biochem.*, **74**, 94.

ULLRICH, V. & KREMERS, P. (1977) Multiple forms of cytochrome P450 in the microsomal monooxygenase system. *Arch. Toxicol.*, **39**, 41.

VAN CANTFORT, J. & GIELEN, J. E. (1975) Organ specificity of aryl hydrocarbon hydroxylase induction by cigarette smoke in rats and mice. *Biochem. Pharmacol.*, **24**, 1253.

VAN CANTFORT, J. & GIELEN, J. E. (1977) Induction by cigarette smoke of aryl hydrocarbon hydroxylase activity in the rat kidney and lung. *Int. J. Cancer*, **19**, 538.

VAN CANTFORT, J., DE GRAEVE, J. & GIELEN, J. E. (1977) Radioactive assay for aryl hydrocarbon hydroxylase. Improved method and biological importance. *Biochem. Biophys. Res. Commun.*, **79**, 505.

VAN CANTFORT, J., MANIL, L., GIELEN, J. E., GLATT, H. R. & OESCH, F. (1979) A new assay for glutathione S-transferase using [$^3$H]-benzo(a)pyrene 4,5-oxide as substrate: Inducibility by various chemicals in different rat tissues compared to that of aryl hydrocarbon hydroxylase and epoxide hydratase. *Biochem. Pharmacol.*, **28**, 455.

VAUGHT, J. B., GURTOO, H. L., PARKER, N. B., LEBOEUF, R. & DOCTOR, G. (1979) Effect of smoking on benzo(a)pyrene metabolism by human placental microsomes. *Cancer Res.*, **39**, 3177.

WEISBURGER, E. K. (1978) Mechanisms of chemical carcinogenesis. *Ann. Rev. Pharmacol. Toxicol.*, **18**, 395.

WELCH, R. M., LOH, A. & CONNEY, A. H. (1971) Cigarette smoke: Stimulatory effect on metabolism of 3,4-benzopyrene by enzymes in rat lung. *Life Sci.*, **10**, 215.

WOLFF, T., DEML, E. & WANDERS, H. (1979) Aldrin epoxidation, a highly sensitive indicator specific for cytochrome P450-dependent monooxygenase activities. *Drug Metab. Dispos.*, **7**, 301.

WYNDER, E. L. & MABUCHI, K. (1972) Etiologica and preventive aspects of human cancer. *Prev. Med.*, **1**, 300.


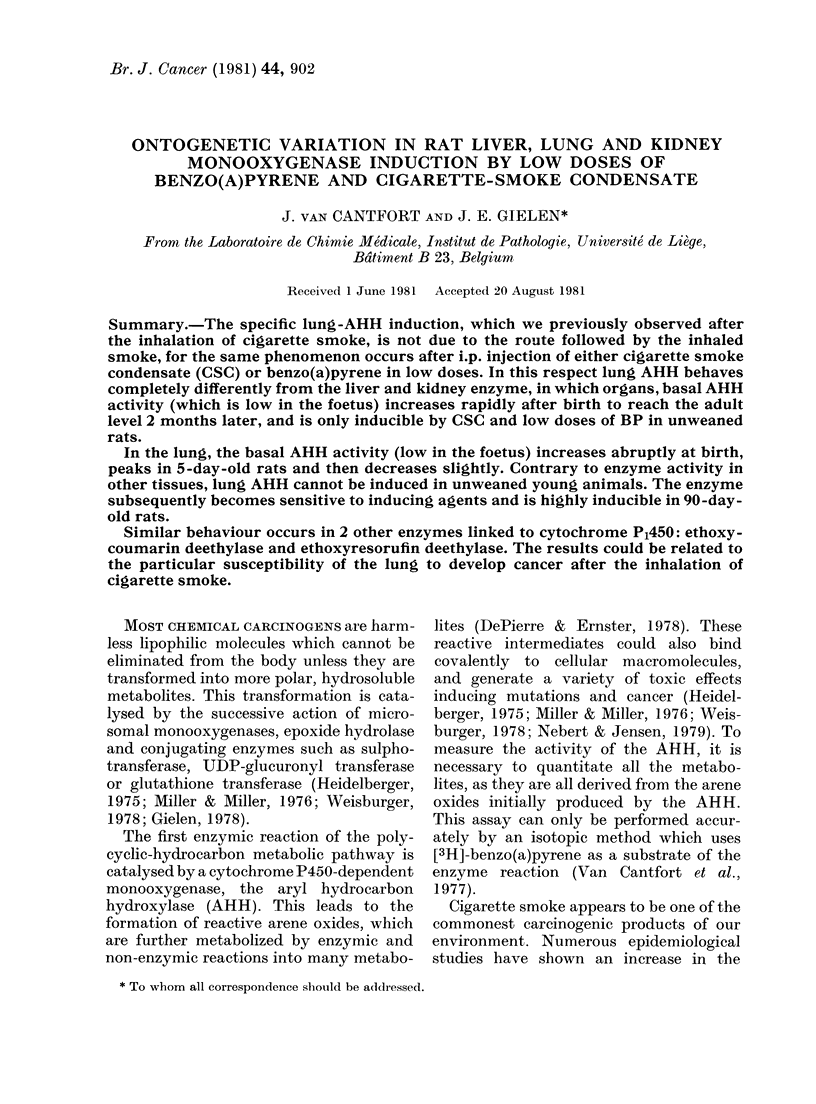

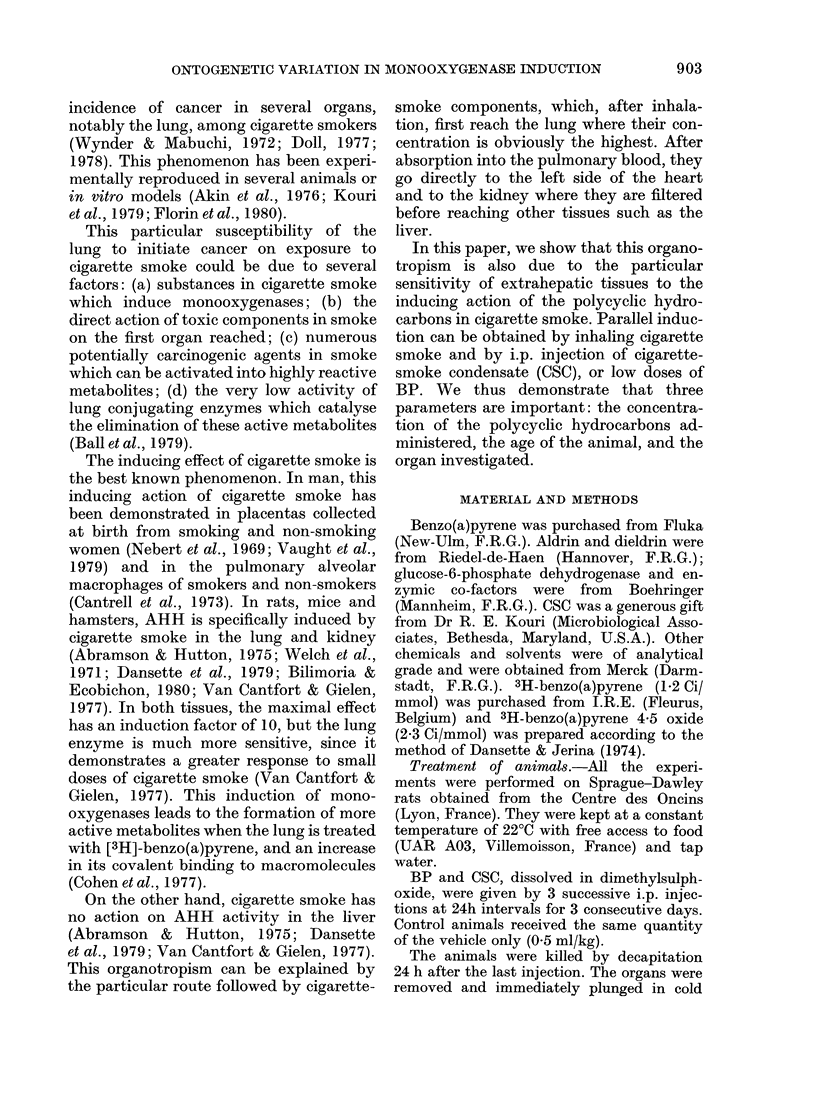

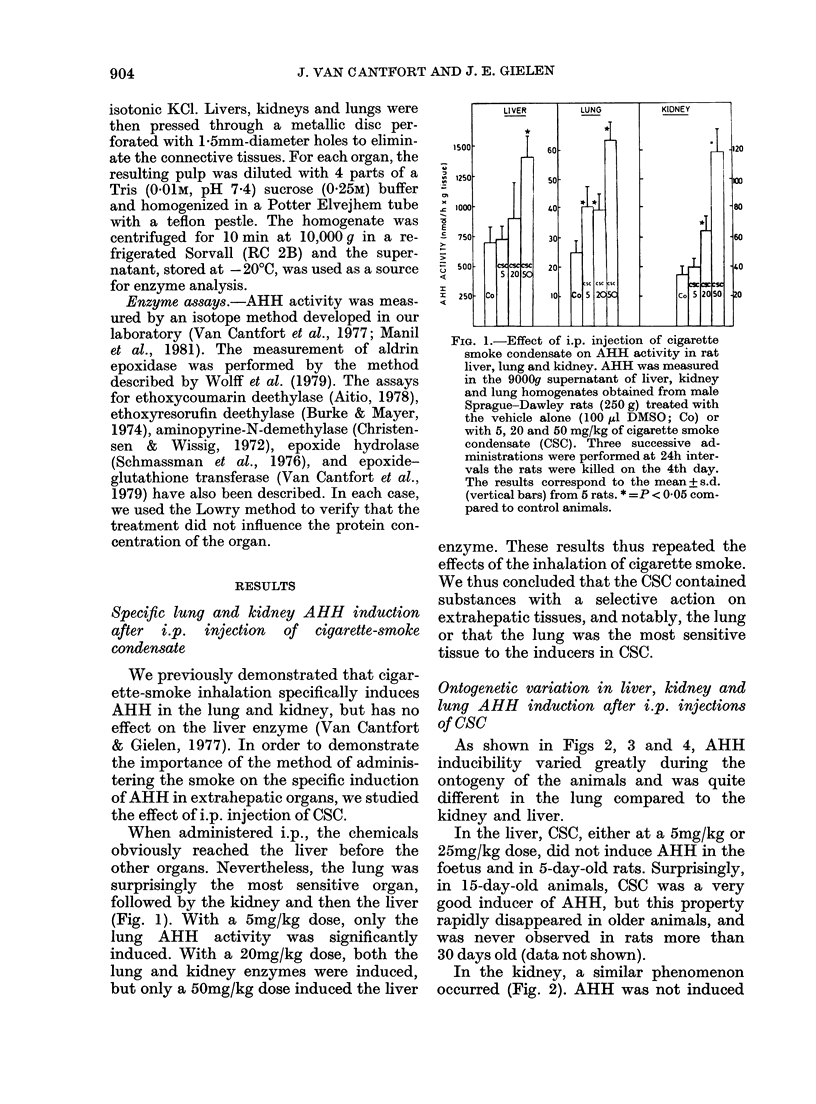

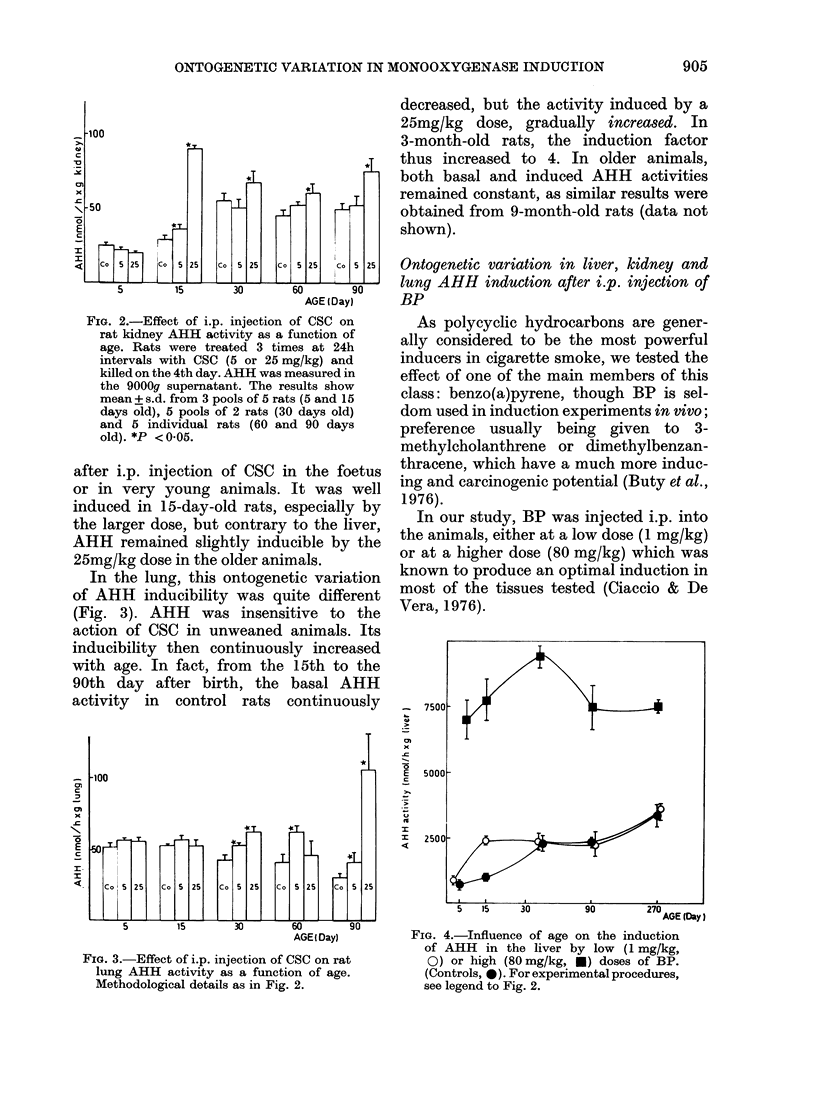

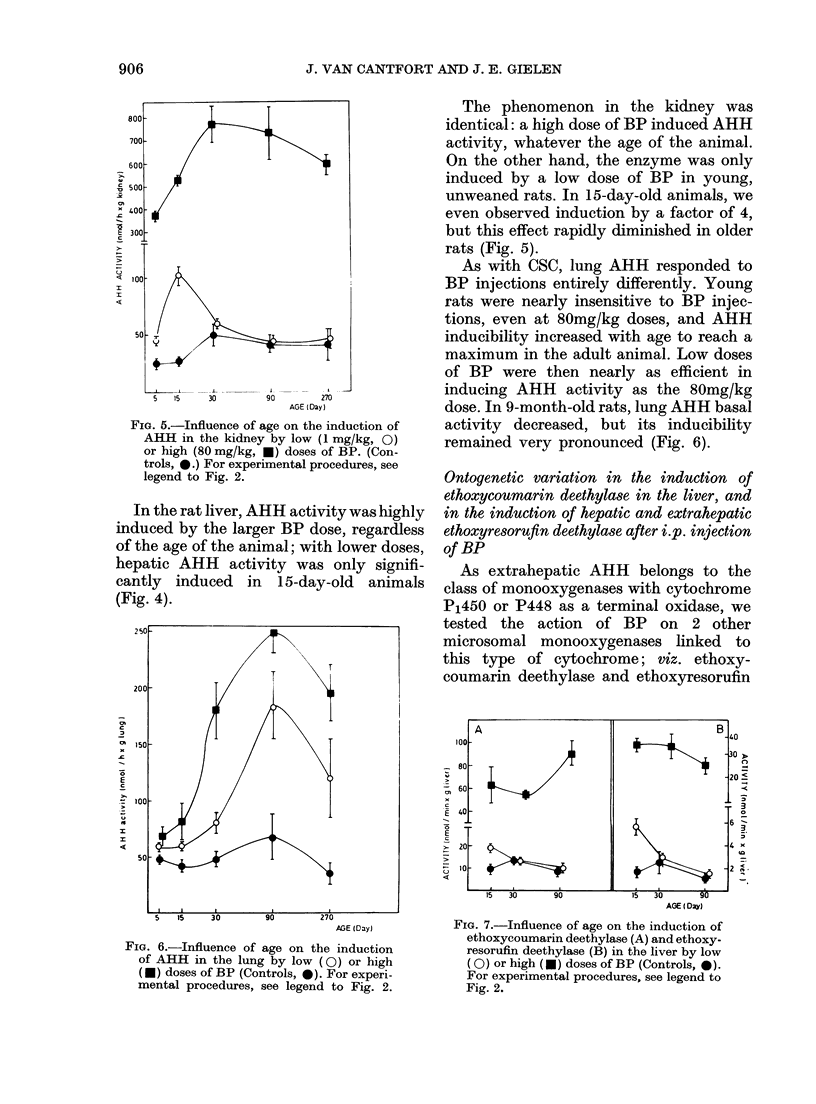

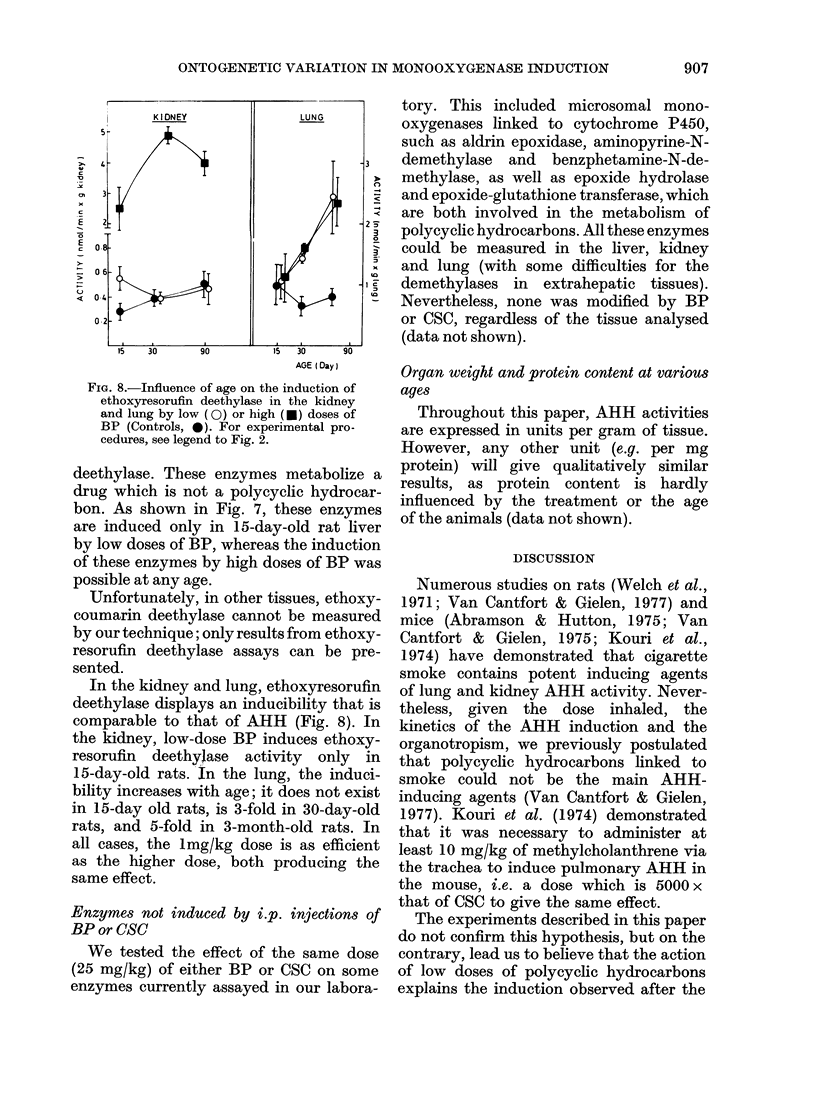

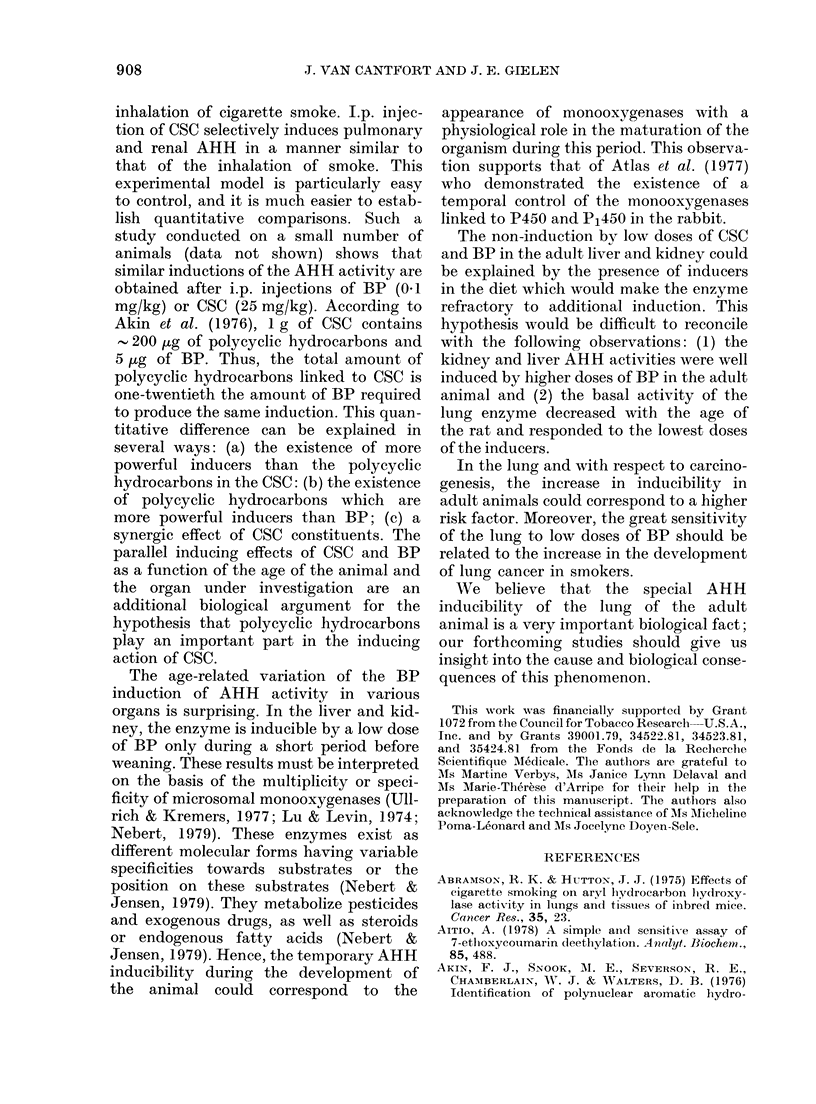

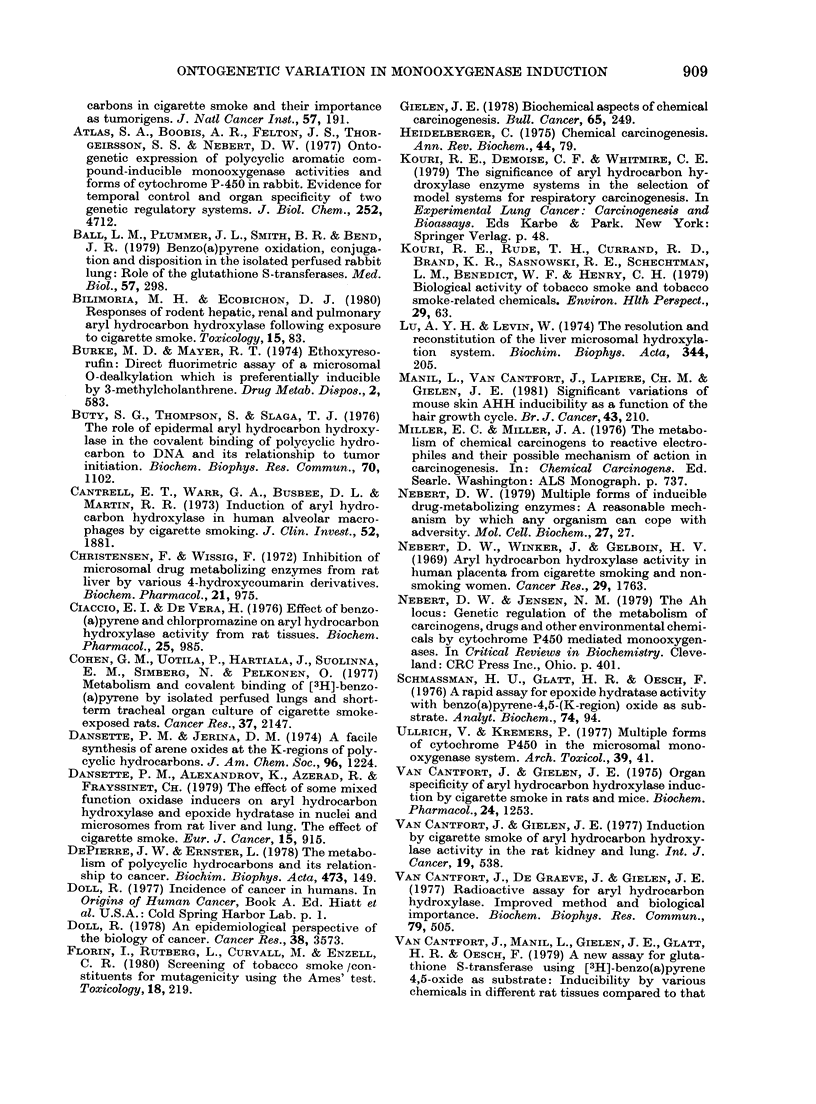

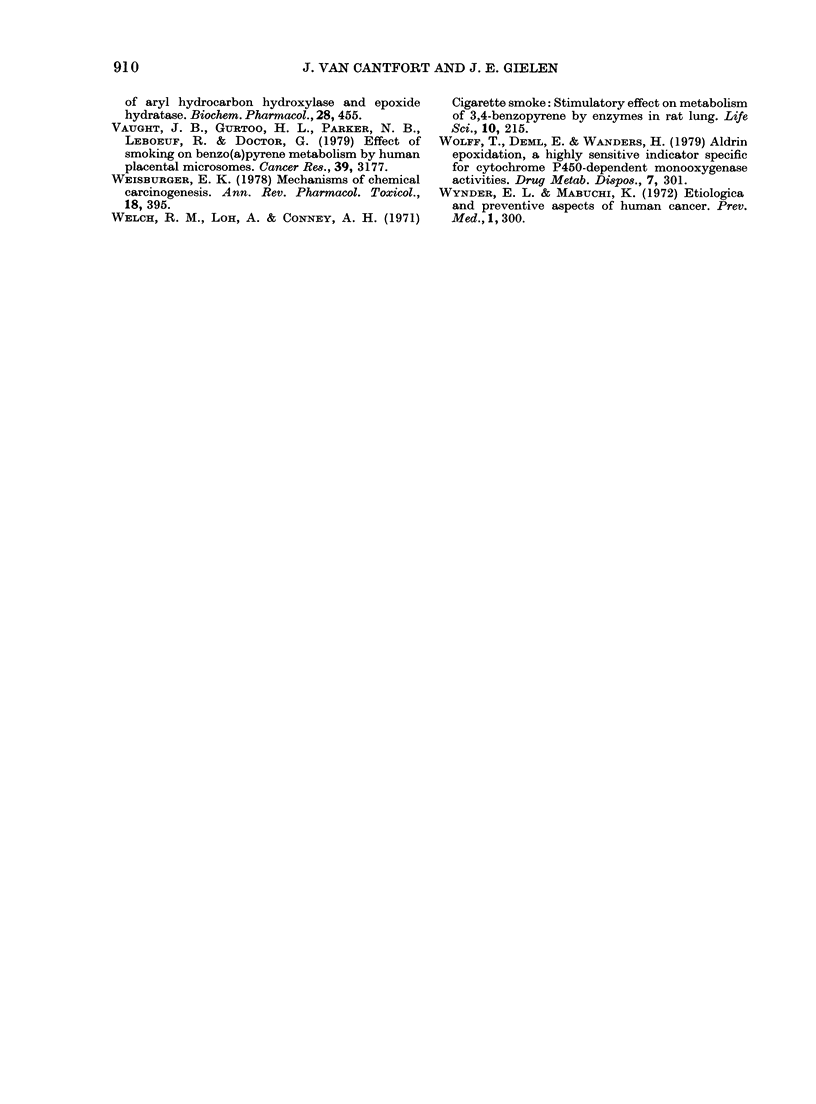


## References

[OCR_00779] Abramson R. K., Hutton J. J. (1975). Effects of cigarette smoking on aryl hydrocarbon hydroxylase activity in lungs and tissues of inbred mice.. Cancer Res.

[OCR_00785] Aitio A. (1978). A simple and sensitive assay of 7-ethoxycoumarin deethylation.. Anal Biochem.

[OCR_00802] Atlas S. A., Boobis A. R., Felton J. S., Thorgeirsson S. S., Nebert D. W. (1977). Ontogenetic expression of polycyclic aromatic compound-inducible monooxygenase activities and forms of cytochrome P-450 in rabbit. Evidence for temporal control and organ specificity of two genetic regulatory systems.. J Biol Chem.

[OCR_00810] Ball L. M., Plummer J. L., Smith B. R., Bend J. R. (1979). Benzo(a)pyrene oxidation, conjugation and disposition in the isolated perfused rabbit lung: role of the glutathione S-transferases.. Med Biol.

[OCR_00817] Bilimoria M. H., Ecobichon D. J. (1980). Responses of rodent hepatic, renal and pulmonary aryl hydrocarbon hydroxylase following exposure to cigarette smoke.. Toxicology.

[OCR_00823] Burke M. D., Mayer R. T. (1974). Ethoxyresorufin: direct fluorimetric assay of a microsomal O-dealkylation which is preferentially inducible by 3-methylcholanthrene.. Drug Metab Dispos.

[OCR_00830] Buty S. G., Thompson S., Slaga T. J. (1976). The role of epidermal aryl hydrocarbon hydroxylase in the covalent binding of polycyclic hydrocarbon to DNA and its relationship to tumor initiation.. Biochem Biophys Res Commun.

[OCR_00838] Cantrell E. T., Warr G. A., Busbee D. L., Martin R. R. (1973). Induction of aryl hydrocarbon hydroxylase in human pulmonary alveolar macrophages by cigarette smoking.. J Clin Invest.

[OCR_00845] Christensen F., Wissing F. (1972). Inhibition of microsomal drug-metabolizing enzymes from rat liver by various 4-hydroxycoumarin derivatives.. Biochem Pharmacol.

[OCR_00851] Ciaccio E. I., De Vera H. (1976). Effect of benzo(a)pyrene and chlorpromazine on aryl hydrocarbon hydroxylase activity from rat tissues.. Biochem Pharmacol.

[OCR_00857] Cohen G. M., Uotila P., Hartiala J., Suolinna E. M., Simberg N., Pelkonen O. (1977). Metabolism and covalent binding of [3H]benzo(a)pyrene by isolated perfused lungs and short-term tracheal organ culture of cigarette smoke-exposed rats.. Cancer Res.

[OCR_00869] Dansette P. M., Alexandror K., Azerad R., Frayssinet C. (1979). The effect of some mixed function oxidase inducers on aryl hydrocarbon hydroxylase and epoxide hydrase in nuclei and microsomes from rat liver and lung. The effect of cigarette smoke.. Eur J Cancer.

[OCR_00865] Dansette P., Jerina D. M. (1974). A facile synthesis of arene oxides at the K regions of polycylic hydrocarbons.. J Am Chem Soc.

[OCR_00877] DePierre J. W., Ernster L. (1978). The metabolism of polycyclic hydrocarbons and its relationship to cancer.. Biochim Biophys Acta.

[OCR_00886] Doll R. (1978). An epidemiological perspective of the biology of cancer.. Cancer Res.

[OCR_00890] Florin I., Rutberg L., Curvall M., Enzell C. R. (1980). Screening of tobacco smoke constituents for mutagenicity using the Ames' test.. Toxicology.

[OCR_00896] Gielen J. E. (1978). Biochemical aspects of chemical carcinogenesis.. Bull Cancer.

[OCR_00900] Heidelberger C. (1975). Chemical carcinogenesis.. Annu Rev Biochem.

[OCR_00913] Kouri R. E., Rude T. H., Curren R. D., Brandt K. R., Sosnowski R. G., Schechtman L. M., Benedict W. F., Henry C. J. (1979). Biological activity of tobacco smoke and tobacco smoke-related chemicals.. Environ Health Perspect.

[OCR_00921] Lu A. Y., Levin W. (1974). The resolution and reconstitution of the liver microsomal hydroxylation system.. Biochim Biophys Acta.

[OCR_00929] Manil L., Van Cantfort J., Lapière C. M., Gielen J. E. (1981). Significant variation in mouse-skin aryl hydrocarbon hydroxylase inducibility as a function of the hair growth cycle.. Br J Cancer.

[OCR_00952] Nebert D. W., Jensen N. M. (1979). The Ah locus: genetic regulation of the metabolism of carcinogens, drugs, and other environmental chemicals by cytochrome P-450-mediated monooxygenases.. CRC Crit Rev Biochem.

[OCR_00940] Nebert D. W. (1979). Multiple forms of inducible drug-metabolizing enzymes: a reasonable mechanism by which any organism can cope with adversity.. Mol Cell Biochem.

[OCR_00946] Nebert D. W., Winker J., Gelboin H. V. (1969). Aryl hydrocarbon hydroxylase activity in human placenta from cigarette smoking and nonsmoking women.. Cancer Res.

[OCR_00960] Schmassmann H. U., Glatt H. R., Oesch F. (1976). A rapid assay for epoxide hydratase activity with benzo (a)pyrene 4,5-(K-region)-oxide as substrate.. Anal Biochem.

[OCR_00966] Ullrich V., Kremers P. (1977). Multiple forms of cytochrome P450 in the microsomal monooxygenase system.. Arch Toxicol.

[OCR_00983] Van Cantfort J., De Graeve J., Gielen J. E. (1977). Radioactive assay for aryl hydrocarbon hydroxylase. Improved method and biological importance.. Biochem Biophys Res Commun.

[OCR_00977] Van Cantfort J., Gielen J. (1977). Induction by cigarette smoke of aryl hydrocarbon hydroxylase activity in the rat kidney and lung.. Int J Cancer.

[OCR_00971] Van Cantfort J., Gielen J. (1975). Organ specificity of aryl hydroxylase induction by cigarette smoke in rats and mice.. Biochem Pharmacol.

[OCR_00990] Van Cantfort J., Manil L., Gielen J. E., Glatt H. R., Oesch F. (1979). A new assay for glutathione S-transferase using [3H]-benzo(a)pyrene 4,5-oxide as substrate. Inducibility by various chemicals in different rat tissues compared to that of aryl hydrocarbon hydroxylase and epoxide hydratase.. Biochem Pharmacol.

[OCR_01002] Vaught J. B., Gurtoo H. L., Parker N. B., LeBoeuf R., Doctor G. (1979). Effect of smoking on benzo(a)pyrene metabolism by human placental microsomes.. Cancer Res.

[OCR_01008] Weisburger E. K. (1978). Mechanisms of chemical carcinogenesis.. Annu Rev Pharmacol Toxicol.

[OCR_01013] Welch R. M., Loh A., Conney A. H. (1971). Cigarette smoke: stimulatory effect of metabolism of 3,4--benzpyrene by enzymes in rat lung.. Life Sci I.

[OCR_01019] Wolff T., Deml E., Wanders H. (1979). Aldrin epoxidation, a highly sensitive indicator specific for cytochrome P-450-dependent mono-oxygenase activities.. Drug Metab Dispos.

[OCR_01025] Wynder E. L., Mabuchi K. (1972). Etiological and preventive aspects of human cancer.. Prev Med.

